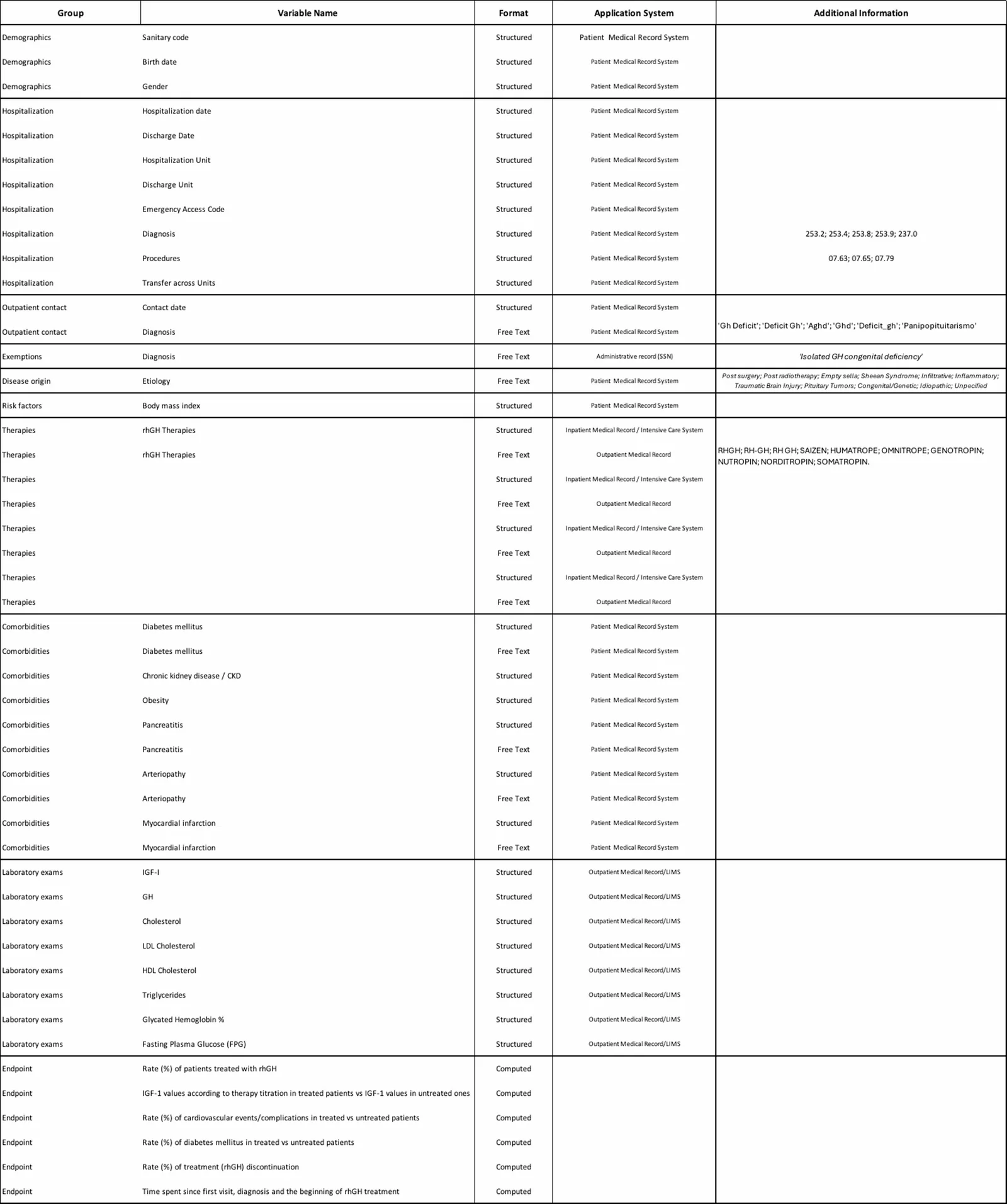# Building the adult growth hormone deficiency data mart: a Real-World model of AI-driven clinical data extraction in a single Italian center

**DOI:** 10.1007/s40618-026-02918-z

**Published:** 2026-05-26

**Authors:** Edoardo Vergani, Chiara Iacomini, Antonella Giampietro, Domenico Milardi, Sabrina Chiloiro, Antonio Mancini, Laura De Marinis, Antonio Bianchi, Stefano Patarnello, Alfredo Pontecorvi

**Affiliations:** 1 Complex Operative Unit of Internal Medicine, Endocrinology and Diabetology, Fondazione Policlinico Universitario Agostino Gemelli IRCCS, Università Cattolica del Sacro Cuore, Rome, Italy; 2 International Scientific Institute Paolo VI, Rome, Italy; 3 Gemelli Generator, Fondazione Policlinico Universitario Agostino Gemelli IRCCS, Rome, Italy

**Keywords:** Artificial intelligence, Growth hormone deficiency, Growth hormone, Data mart, Real world data, Real world evidence, Natural language processing

## Abstract

**Purpose:**

Adult Growth Hormone Deficiency (AGHD) is a complex and under-recognized condition, mainly managed in outpatient settings and characterized by fragmented and heterogeneous clinical data. Population registries provide high-quality evidence but require long timeframes and substantial resources. The aim of this study was to design and validate an artificial intelligence (AI)-driven methodology for the construction of a disease-specific AGHD Data Mart from routine clinical data within a single high-volume center, to support real-world evidence generation and future advanced analytics.

**Methods:**

A standardized Data Science framework, based on automated extraction from hospital data warehouses and electronic medical records, integrating structured data and unstructured clinical narratives through natural language processing, was implemented. AGHD patients were identified using a combination of ICD-9 codes and text-mining applied to outpatient reports. A multidisciplinary workflow ensured clinical validation of extracted data. The Data Mart described patient identification from first hospital access (T0) and included diagnostic modality, etiology, biochemical data, comorbidities, and growth hormone replacement therapy.

**Results:**

Among 210 identified patients, 188 were validated as AGHD after expert review. Diagnoses were based on dynamic testing (28.2%), panhypopituitarism with low IGF-1 (54.3%), or AI-assisted identification (17.6%). Etiology was retrieved in 87.8% of cases, with post-surgical causes being the most frequent. 37.8% of patients were receiving rhGH therapy. Specific trends for IGF-1 values for single patients were described.

**Conclusion:**

This study represents the first AI-driven AGHD Data Mart and demonstrates the feasibility of constructing the Data Mart from routine clinical data. This approach offers a complementary framework for structured RWD extraction and may support future longitudinal analyses and AI-based clinical support in AGHD.

## Introduction

Adult Growth Hormone Deficiency (AGHD) represents a complex endocrine disorder characterized by insufficient secretion of growth hormone from the pituitary gland, which can lead to an unfavorable metabolic profile (increased visceral adiposity, reduced lean mass and decreased bone density), and quality of life impairment (fatigue, mood disturbances, and cognitive impairment) [1]. Its clinical manifestations are subtle and may overlap with metabolic syndrome or depression; moreover, it can occur either isolated or within the broader context of hypopituitarism. Replacement therapy with recombinant human growth hormone (rhGH) aims to improve body composition, reduce symptom burden, and enhance quality of life [2, 3].

Although clinical and research progress has been significant, important unmet needs persist [4]. The transition from pediatrics to adult care is still an open issue [5]. Long-term data confirming the durability of GH replacement benefits is lacking. The diagnostic variability, the absence of validated biomarkers (other than Insulin-like Growth Factor - IGF-1), and the need for better patient stratification continue to complicate clinical decision-making [4]. Furthermore, tailoring GH therapy to individual characteristics such as phenotype, comorbidities, and response to treatment is not yet routine in clinical practice [2]. Finally, suboptimal adherence remains another major limitation, highlighting the relevance of patient engagement and digital monitoring tools [6].

Most current knowledge on the “real-life” management of AGHD derives from large-scale observational registries, including ANSWER [7], NordiNET [7], KIMS [8], PATRO Adults [9], and the HypoCCS [10]. These databases have brought important insights into the real-world use of somatropin and its outcomes. However, such projects are resource-intensive and require long observation periods (often decades) to produce operational evidence [11].

Data Science, which includes advanced data processing methods and extensive use of Artificial intelligence (AI) techniques, represents an opportunity to address some of these challenges through the acceleration of insights generation from real-world data (RWD) [12]. Unlike traditional registries, RWD, derived from electronic medical records (EMR), claims databases, and other routine care sources, can capture a broader, more dynamic picture of clinical practice, that can enrich the precious and accurate data coming from registries [13].

All the works dealing with RWD present methodological complexities, given the difficult curation of large datasets with the need for predefined data fields, without neglecting the substantial financial and technical resources needed. To overcome these obstacles, AI-driven analytics can extract, integrate, and interpret data efficiently, transforming unstructured clinical information into meaningful Real World Evidence (RWE) [14].

In this context, the Fondazione Policlinico Universitario A. Gemelli IRCCS (Rome, Italy) has established the Gemelli Generator Real World Data Facility, a multidisciplinary research facility with the aim of deriving clinically meaningful evidence from clinical RWD [15]. The facility integrates methods of data harmonization, visualization, and AI-supported predictive modeling. The Gemelli Generator Data Science framework validates a structured information architecture for RWE generation. Data from heterogeneous clinical sources, such as hospitalizations, diagnoses, therapies, laboratory results, and patient-reported outcomes, are integrated through standardized ontologies and continuous extraction processes [15]. According to the above-mentioned flow, structured Data Warehouses and IT Application Hospital systems are the input for disease-specific Data Marts, enabling longitudinal, continued, patient-centered repositories. Data marts are the backbone of a system that, through AI methods (pattern recognition, natural language processing, and large language models), enable multimodal data integration and advanced analytics. This hierarchical approach allows the transformation of raw clinical data into interpretable, predictive, and practical evidence across multiple disease domains [16, 17]. This framework has already been applied successfully to oncology fields such as breast cancer [18] and other medical fields such as infective diseases, stroke, dyslipidemia, systemic lupus, heart failure [19–23]; however, there is no experience in the endocrinology field.

Accordingly, the aim of our study was to design and validate a methodology capable of constructing an AGHD-specific Data Mart from a single high-volume Italian center through the use of AI systems, specifically automated extraction from EMR, and natural language processing (NLP). This is a methodological study with illustrative clinical outputs. This Data Mart is intended to provide an accurate description of the real-world AGHD population, to identify clinical and management biases, and to lay the basis for future RWE studies applying large language models (LLMs) and machine learning approaches in GHD.

## Methods

A retrospective observational study was conducted following the principles of the Helsinki Declaration (2013 revision). The study protocol was reviewed and approved by the Institutional Review Board of the Ethical Committee of “Lazio area 3” (protocol number: 7384 – approved on 06/03/2025).

### General concepts

The Data Science Laboratory at Policlinico Gemelli has implemented a standardized methodology to collect and organize data from routine care processes, targeted at RWE generation. This is a comprehensive library of data processing procedures to deliver in-depth representations of patients’ disease trajectories over time. The procedures are organized with a layered sequence of steps: (a) extraction, transformation and load (ETL) from hospital operational systems/data warehouse; (b) creation of the disease-specific integrated datasets; (c) analytics and descriptive models.

The longitudinal disease-specific repositories generated are indexed through individual patient pseudonymized identifier (i.e. patient-centered) enabling clinical researchers to perform their analyses by navigating effectively across subsets of the overall population (based on study-specific inclusion criteria) or individual cases which require special attention. These structured, disease-specific datasets are defined as the Gemelli ‘Disease Data Marts’ and made available to the clinical community to address their research questions.

The approach covers data processing in different formats: structured data are directly extracted from administrative databases, laboratory systems, electronic medication administration records; unstructured data (e.g. clinical diaries and reports, outpatients follow up visits, discharge letters) were processed using clinically validated NLP algorithms and eventually LLM, dealing with semantic variations and experts’ domain dictionaries as they occur in free-text documents. All extracted data were standardized and mapped to common data elements within the Disease Data Mart to ensure consistency and interoperability. Validation procedures were applied to a random sample of records to confirm the accuracy of ETL pipelines and NLP/LLM procedures. In terms of technology, the Data Science framework makes use of:

- SAS(R) v. 9.04 is used as a middleware for ETL tasks from hospital systems and DWH, as well as for NLP procedures. The processed data are stored in structured tables within Microsoft SQL Server 16.0, organized in dedicated storage areas for downstream analyses.
- Python v.3.14.0 and R v.4.2.1 are used for data analytics, data processing, data visualization, and modelling activities.

In our study, the AGHD Data Mart was conceived as a specified, clinically oriented repository derived from the broader Gemelli hospital data infrastructure. Consistently with the general framework, the AGHD Data Mart is the curated subset of the institutional Data Warehouse, centered on this disease domain and designed to provide an integrated view of patients’ clinical histories, laboratory data, biomarkers, therapies, and outcomes - the dictionary of relevant healthcare data and associated clinical validation constitutes the ontology of this disease Datamart. Since AGHD is mainly an outpatients’ service disease, the main source of information is patients’ EMR, decoded through NLP techniques, text mining and pattern matching supervised by clinicians.

The AGHD Data Mart contains longitudinal and continuously updated clinical information for all adult patients with a confirmed diagnosis of GHD. Each clinical contact, whether outpatient visits, day-hospital evaluations, or admissions, is captured and harmonized into the repository. As a first step, in this manuscript, we deliberately focused on the first foundational component of the framework: the identification of patients with AGHD at their first point of contact within our university hospital system. This step was essential to ensure a reliable and reproducible definition of the cohort before expanding the architecture to longitudinal follow-up and advanced analytics. Consequently, the dataset includes a selected group of clinically meaningful variables such as the confirmed diagnosis of GHD, the diagnostic pathway leading to it, prescribed GH replacement therapy, relevant biochemical results, age at presentation, and other key demographic or clinical attributes. This approach allowed us to establish a robust baseline against which future longitudinal developments of Data Mart can be reliably built.

### Multidisciplinary approach - clinicians and data scientists’ interaction

The methodological framework aligns with contemporary standards for high-quality RWD studies, including the principles of CODE-EHR initiative. Dataset building and linkage procedures, together with ontology definition, coding rules, and outcome description, follow standardized internal protocols of the Gemelli Generator Real World Data Facility. All activities were developed within a governance structure fully compliant with GDPR and national regulations, under the supervision of the institutional Data Protection Officer. Data visualization and analytics are performed using the technology suite described above.

The framework is designed to be regularly updated with new data and new patients. The new data related to patients already included can be updated daily when a new contact occurs. New patients meeting the inclusion criteria may be included in the Data Mart on regular basis.

A central element of this project was the continuous and structured interaction between clinicians, data scientists, and data analysts. Since the early stages of the study, where the focus was mainly on methodological steps, data extraction strategies, and preliminary results, a close interaction among clinical and technical expertise was established through regular in person and online meetings. These sessions allowed both sides to progressively align on a shared terminology, ensuring that clinical needs, operational constraints, and analytical methods were clearly understood by all team members. Informal exchanges and follow-up discussions were also encouraged whenever specific issues emerged, helping to maintain a smooth workflow and to promptly address ambiguities in variable definitions or model interpretations. By doing so, continuous improvement on dataset generation and intermediate results validation from clinicians was put in place. This collaborative approach, based on iterative feedback and mutual adaptation, proved essential for building a coherent Data Mart.

The general validation procedures described above were applied to ensure consistency and accuracy across all transformed variables. Structured and computed fields were reviewed by data scientists and clinicians, who examined their distributions and acceptable value ranges before finalizing the corresponding ETLs.

For unstructured data, extracted variables underwent an initial technical validation, followed by independent clinical review. Evaluation reports generated throughout this process supported the iterative refinement of text-mining rules. Finally, for variables expected to maintain internal coherence (such as BMI and obesity), we verified that logical relationships were preserved as an indicator of data consistency.

## Results

### Population definition and extraction

**Fig. 1:**
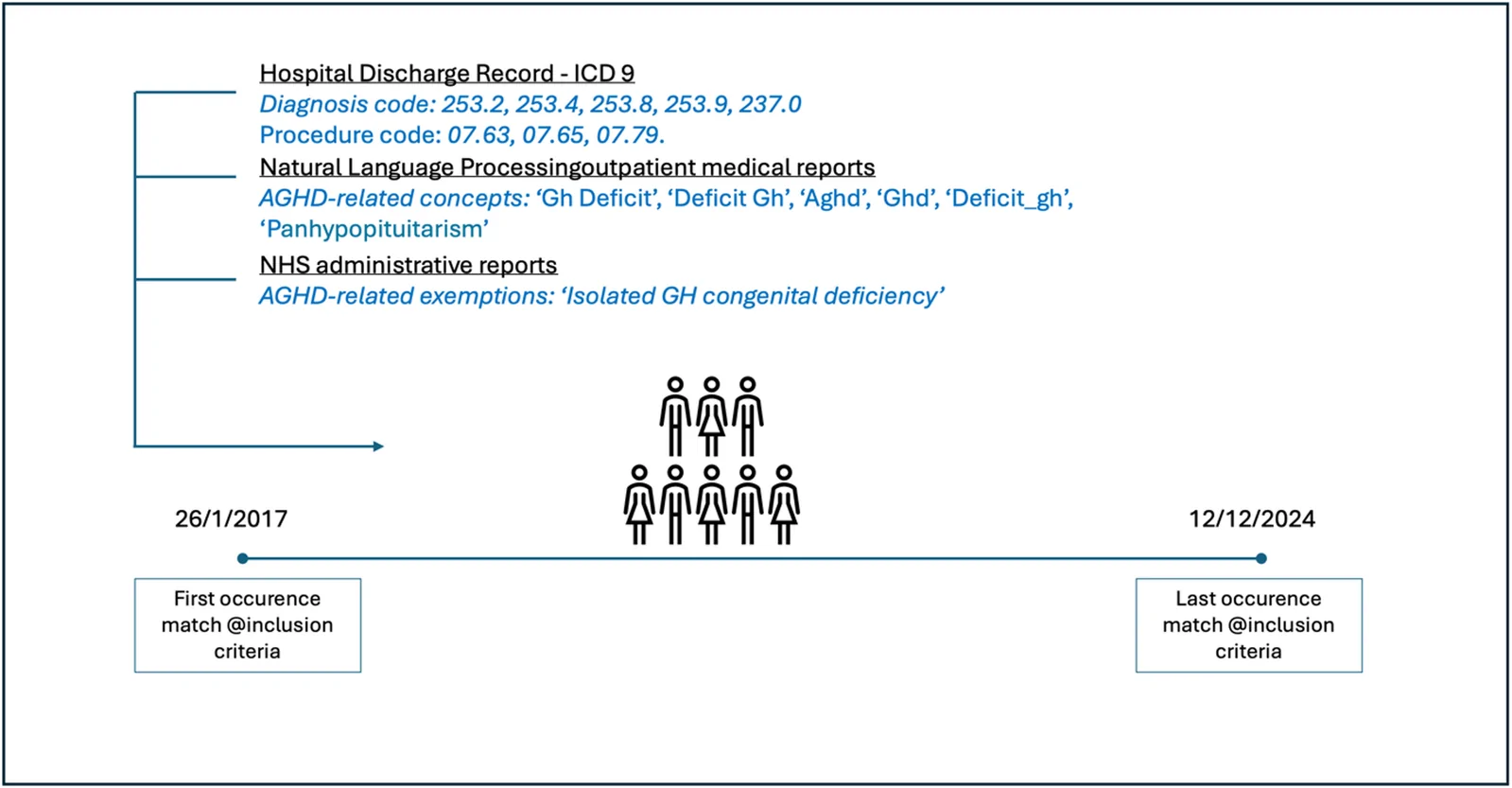
Extraction process. The image shows a brief and schematic overview of the extraction process

The development of the AGHD Data Mart followed the general philosophy of the GENERATOR framework [15], which is based on close collaboration between clinicians familiar with the disease, data scientists, and data analysts. As with all domain-specific Data Marts, the process began with the definition by the clinicians of clear inclusion criteria to detect the studied population. The inclusion criteria were patients over 18 years and diagnosis of GHD, according to the current guidelines (Fig. 1).

To ensure comprehensive patient capture, we used four complementary inclusion strategies:

1. Hospital Discharge Forms (HDF)-based identification: Selection through ICD-9 codes recorded in hospital discharge forms, including diagnoses *253.2*,* 253.4*,* 253.8*,* 253.9*,* 237.0* and procedures *07.63*,* 07.65*,* 07.79*.
2. HDF excluding code 237.0: A more restrictive identification using only the 253.x diagnostic series and the same procedures.
3. Text-mining of outpatient reports (TrakCare^®^): Through NLP, AGHD-related concepts were extracted from endocrinology outpatient notes.
4. Exemption registry: Screening for the specific exemption “Isolated GH congenital deficiency”.

The time window for diagnosis in this cohort ranged from January 26, 2017, to December 12, 2024.

**Fig. 2:**
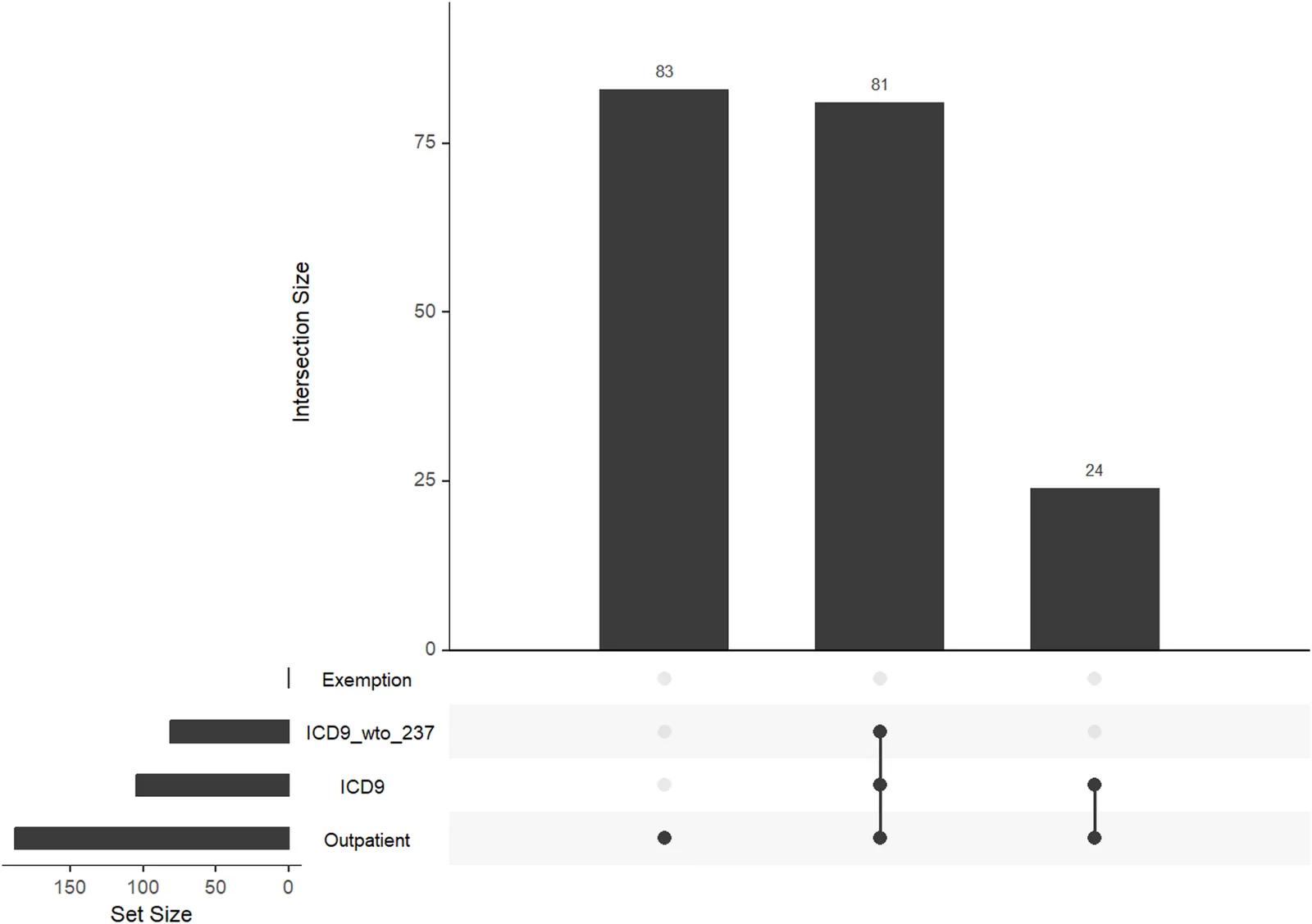
AGHD patients’ extraction. The figure shows the distribution of the cohort with respect to the different identification criteria for inclusion of the patients in the Data Mart. Exemption: Exemption registry; ICD9_wto_237: HDF excluding code 237.0; HDF-based identification: ICD9; Text-mining: Outpatient.

Based on these indications, the data science team executed the first extraction step.

The identification of AGHD patients was established using a text-mining approach applied to outpatient clinical reports. The procedure aimed to systematically capture any narrative evidence compatible with an explicit diagnosis of adult-onset growth hormone deficiency. A keyword dictionary was developed to identify diagnostic terminology associated with AGHD. The following inclusion terms were queried across all available unstructured reports: GH DEFICIT, DEFICIT GH, AGHD, GHD, DEFICIT_GH, PANHYPOPITUITARISM and HYPOPITUITARISM. Since through such criteria reports describing growth hormone deficiency in pediatric contexts could also be selected, the acronym PGHD (Pediatric Growth Hormone Deficiency) and mention of the term ‘pediatric’ in clinical notes were explicitly defined as exclusion criteria.

The presence of either exclusion term automatically removed the patient from eligibility for the AGHD cohort.

To further strengthen the validity of the text-mining–based case identification, an additional validation step was implemented for patients in whom panhypopituitarism was identified in the clinical narratives. Given the well-established association between panhypopituitarism and severe growth hormone deficiency, these patients represent a clinically meaningful subgroup for diagnostic consistency checks. For all patients flagged as having panhypopituitarism through the keyword-based text-mining procedure, laboratory data were queried to assess the presence of insulin-like growth factor 1 (IGF-1) measurements. IGF-1 values were used as a biochemical surrogate marker of growth hormone activity. An IGF-1 value ≤ 75 ng/mL was considered indicative of growth hormone deficiency in patients affected by anterior panhypopituitarism. This serves as an operational proxy.

The validation process consisted of verifying whether patients with narrative evidence of panhypopituitarism also exhibited at least one IGF-1 measurement at or below this threshold. The concordance between textual diagnostic evidence and biochemical findings was evaluated descriptively. The presence of low IGF-1 levels in this subgroup was interpreted as supporting the biological plausibility and clinical accuracy of the text-mining–derived AGHD classification.

A patient was classified as having AGHD if at least one outpatient report contained any of the inclusion keywords and no pediatric-specific exclusion terms. This pattern-based text-mining strategy ensures sensitive and reproducible identification of AGHD diagnoses, especially in clinical settings where diagnostic information may be inconsistently coded or only available in narrative form.

This initial pull led to selecting 210 adult patients with at least one potential indicator of GH deficiency, out of several thousands of patients analyzed from the hospital Data Warehouse using automated procedures. Following a structured validation process performed by the involved endocrinologists, 188 patients were confirmed as eligible for inclusion. The 22 dropped cases after experts’ assessment were associated with incomplete or not definitive diagnosis in the clinical notes, that have been identified from direct human inspection; this means that, though in relatively low occurrences, automated methods should be further improved to capture such nuances (see reference in Discussion section). For confirmed validated occurrences, the diagnosis was defined as follows: assessment of IGF-1 deficiency in case of 3 confirmed pituitary hormonal deficiencies; dynamic test (GHRH + arginine test; insulin-induced hypoglycemia test, glucagon stimulation test) according to the current guidelines [2, 24].

The above-mentioned information was derived from EMR or through the evaluation of the dynamic test recorded on TrakCare^®^.

The extracted data were pseudonymized.

## Variables definition

After that, a preliminary list of clinical variables considered essential for characterizing the population, such as the modality through the diagnosis of GHD has been carried out, biochemical markers, therapy, demographics and comorbidities, was defined by clinicians. Data scientists performed the mapping of all variables across the hospital’s subsystems, mainly EMR (unstructured data) and laboratory platforms (structured data), when possible. The following information were analyzed:

- Demographics (Age, Gender).
- BMI.
- GHD etiologies.
- Type of diagnosis: assessment of IGF-1 deficiency in case of 3 confirmed pituitary hormonal deficiencies vs. dynamic test.
- RHGH Therapy.
- Comorbidities.
- Lab tests: IGF-1, Total Cholesterol, Triglycerides, HDL and LDL Cholesterol, blood Glucose, Glycated Hemoglobin (HBA1c) and Hemoglobin.

To identify exposure to growth hormone replacement therapy, a dedicated text mining procedure was developed and applied to outpatient medical records.

Patients were classified as receiving rhGH therapy if any documented evidence of treatment was identified in their narrative outpatient notes, irrespective of whether such documentation occurred temporally close to the formal AGHD diagnosis. This choice reflects the aim of capturing the patient’s therapeutic history comprehensively, rather than restricting classification to the diagnostic time window alone.

To ensure sensitive and reproducible detection, we defined a structured keyword dictionary encompassing both the active compound and the most commonly prescribed commercial formulations of rhGH. The following lexical patterns were queried across all available unstructured reports: #RHGH, #RH-GH, #RH GH, #SAIZEN, #HUMATROPE, #OMNITROPE, #GENOTROPIN, #NUTROPIN, #NORDITROPIN, #SOMATROPIN.

This pattern-recognition approach enables systematic identification of treatment exposure while mitigating the inherent variability of free-text clinical documentation. Furthermore, by leveraging non-structured data, the method complements traditional structured data extraction and reduces misclassification of rhGH-treated patients that may arise from incomplete or inconsistent coding practices.

A summary table describing the data model used for the study is provided in the Appendix 1.

### Qualitative information analysis

The qualitative analysis of the etiology of AGHD was performed based on the classification reported in the data mart. Eleven main etiological groups were identified:

- Post-surgical.
- Following radiotherapy.
- Empty sella.
- Sheehan Syndrome.
- Infiltrative.
- Inflammatory.
- Traumatic Brain Injury.
- Pituitary Tumors.
- Congenital / Genetic / Structural.
- Idiopathic.
- Unspecified.

**Fig. 3:**
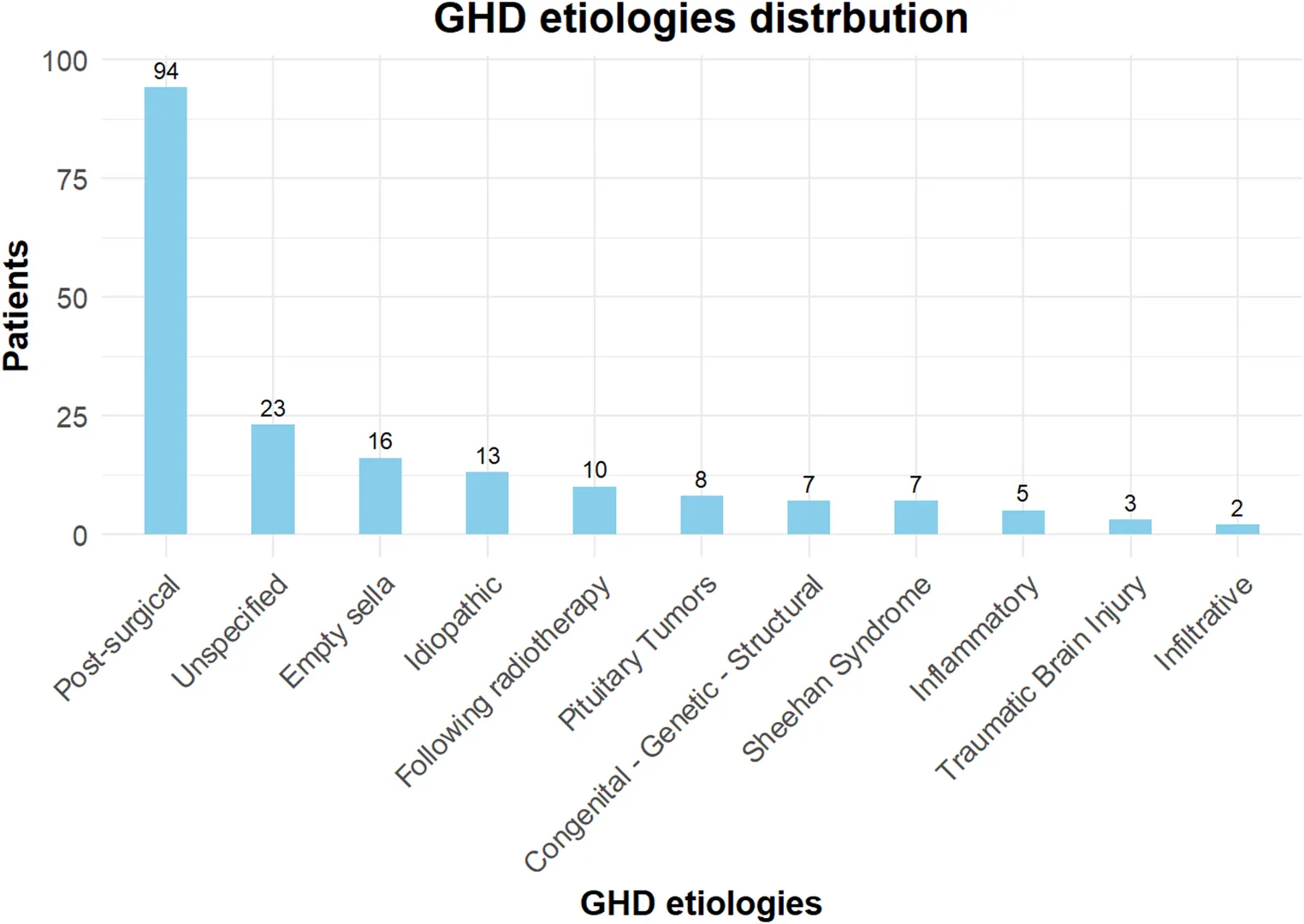
GHD etiologies distribution

The most frequent etiology (Fig. 3) in the cohort was post-surgical (50%), followed by unspecified forms (12.2%), conditions such as empty sella (8.5%), pituitary tumors (4.3%), idiopathic forms (6.9%), and Sheehan syndrome (3.7%). The category “Idiopathic” represent a well-known category in AGHD and our patients were considered idiopathic when specified in the report or after confirmation by the clinician. The category “Unspecified” gathers patients for which a diagnosis of AGHD was specified in the report or in the EMR, but the etiology was not.

Given the presence of a written diagnosis of AGHD in the EMR or EHR of the patient, the modality through which it was carried out was defined based on the origin of clinical identification and the elements available in the clinical reports. Three main modalities were identified:

- Dynamic Test (GHRH stimulation test or access to DH Endocrinology to perform a stimulation test).
- Diagnosis of panhypopituitarism with low IGF-1 confirmed in subsequent reports.
- AI-assisted extraction (automatic extraction via text mining from outpatient reports with no data on the two above-mentioned modality). This last definition refers to patients in whom AGHD was explicitly reported in clinical documentation despite the absence of structured baseline diagnostic data. This typically occurred in cases where objective information, such as IGF-1 values at T0 or results of dynamic testing, was unavailable (i.e. dynamic tests performed elsewhere), but AGHD diagnosis was clearly stated in the medical report.

**Fig. 4:**
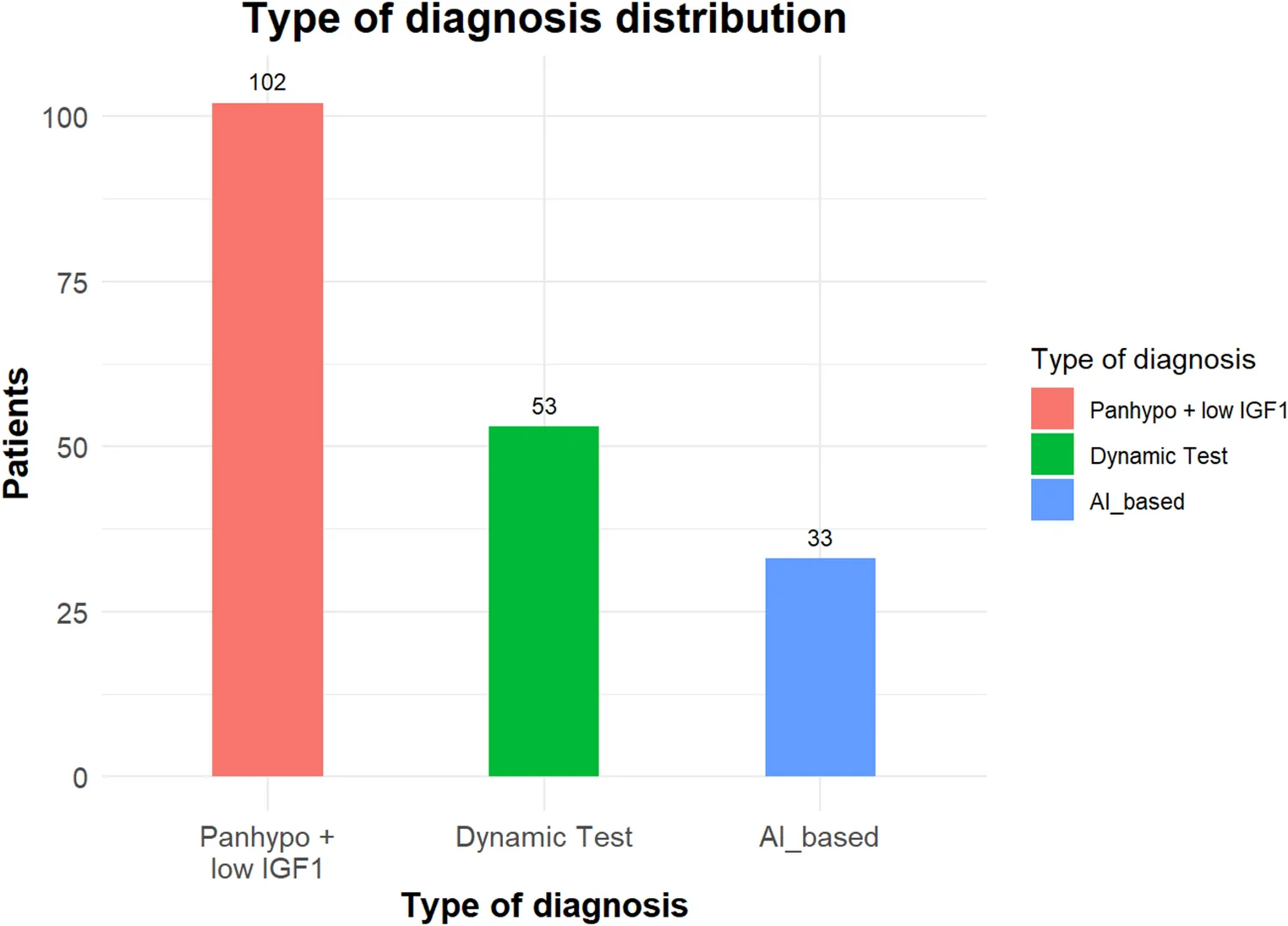
Modality of AGHD diagnosis

The distribution observed in the valid cohort (*N* = 188) shows that (Fig. 4):

- Panhypopituitarism + low IGF-1 contributes to most diagnoses (54.3%).
- Dynamic testing is the second most frequent modality (28.2%).

AI-assisted identification accounts for 17.6% of total diagnoses.

## Quantitative information analysis

This section presents the quantitative results relating to the main biomarkers observed in the population affected by AGHD, considering the tests collected up to ± 365 days from diagnosis.

Both structured (Gemelli laboratory) and unstructured (text mining) values were extracted. The biomarkers included are blood glucose, IGF-1, hemoglobin, HbA1c, total cholesterol, triglycerides, HDL, and LDL.

Table 1 summarizes the mean, median, and range values for each test.

**Table 1:** Overview of laboratory test in AGHD Data Mart

Lab_Test	Mean	Median	Min	Max	*N*	Missing %
Blood Glucose	93,76	84,00	48,00	300,00	119	36,70
Glycated Hemoglobin_Perc	5,63	5,60	3,00	7,40	34	81,91
Glycated Hemoglobin	39,88	37,00	23,00	65,00	33	82,45
Total Cholesterol	199,50	194,38	112,00	374,00	64	65,96
Triglycerides	132,42	118,00	1,25	369,00	67	64,36
HDL Cholesterol	55,36	54,34	28,00	107,00	62	67,02
LDL Cholesterol	118,38	120,00	66,00	227,00	44	76,60
IGF-1	95,95	86,00	21,00	352,00	87	53,72
Hemoglobin	13,16	13,60	8,10	16,10	103	45,21

In the overall cohort (*N* = 188), comprising 117 untreated patients and 71 individuals receiving growth hormone replacement therapy, no significant differences were observed in the main demographic or biochemical characteristics. Mean age did not differ substantially between groups (53.0 ± 18.6 years in untreated patients vs. 49.1 ± 15.8 years in treated patients; *p* = 0.142), and sex distribution was comparable (46.8% female in the total cohort; *p* = 1.000).

Although the distribution of comorbidities showed some non-significant but notable trends—such as a higher prevalence of diabetes in the untreated group and slightly lower rates of metabolic syndrome and dyslipidemia among treated patients—none of these differences reached statistical significance in the comparisons reported.

With regard to metabolic and cardiovascular biomarkers, no statistically significant variations were detected between groups. Mean blood glucose levels were similar (93.6 ± 33.9 mg/dL in untreated patients vs. 94.1 ± 38.0 mg/dL in treated patients; *p* = 0.934), as were total cholesterol (199.8 ± 47.5 vs. 199.2 ± 35.1 mg/dL; *p* = 0.954), HDL cholesterol (57.1 ± 15.8 vs. 53.7 ± 16.3 mg/dL; *p* = 0.414), and LDL cholesterol (115.2 ± 24.2 vs. 121.0 ± 34.8 mg/dL; *p* = 0.531).

Overall, the descriptive analysis indicates that, within the ± 365-day window surrounding the AI diagnosis, laboratory biomarkers and clinical features did not significantly differ between patients receiving and not receiving rhGH (Table 2).

**Table 2:** Descriptive analysis stratified by Target Therapy

Variable	level	Overall	Target Therapy		*p*
			0	1	
n		188	117	71	
Gender (%)	F	88 (46.8)	55 (47.0)	33 (46.5)	1.000
	M	100 (53.2)	62 (53.0)	38 (53.5)	
Age (mean (SD))		51.55 (17.62)	53.03 (18.58)	49.13 (15.75)	0.142
No Comorbidities (mean (SD))		1.89 (1.76)	1.99 (1.79)	1.73 (1.72)	0.330
Dyslipidaemia (%)	1	35 (18.6)	20 (17.1)	15 (21.1)	0.620
Obesity (%)	1	41 (21.8)	27 (23.1)	14 (19.7)	0.720
Heart Disease (%)	1	79 (42.0)	49 (41.9)	30 (42.3)	1.000
Hypertension (%)	1	50 (26.6)	33 (28.2)	17 (23.9)	0.638
Vascular Disease (%)	1	3 ( 1.6)	2 ( 1.7)	1 ( 1.4)	1.000
Diabetes (%)	1	39 (20.7)	30 (25.6)	9 (12.7)	0.052
Metabolic Syndrome (%)	1	94 (50.0)	62 (53.0)	32 (45.1)	0.367
MCE (%)	1	6 ( 3.2)	4 ( 3.4)	2 ( 2.8)	1.000
Lung Disease (%)	1	9 ( 4.8)	6 ( 5.1)	3 ( 4.2)	1.000

## Trends evaluation

**Fig. 5:**
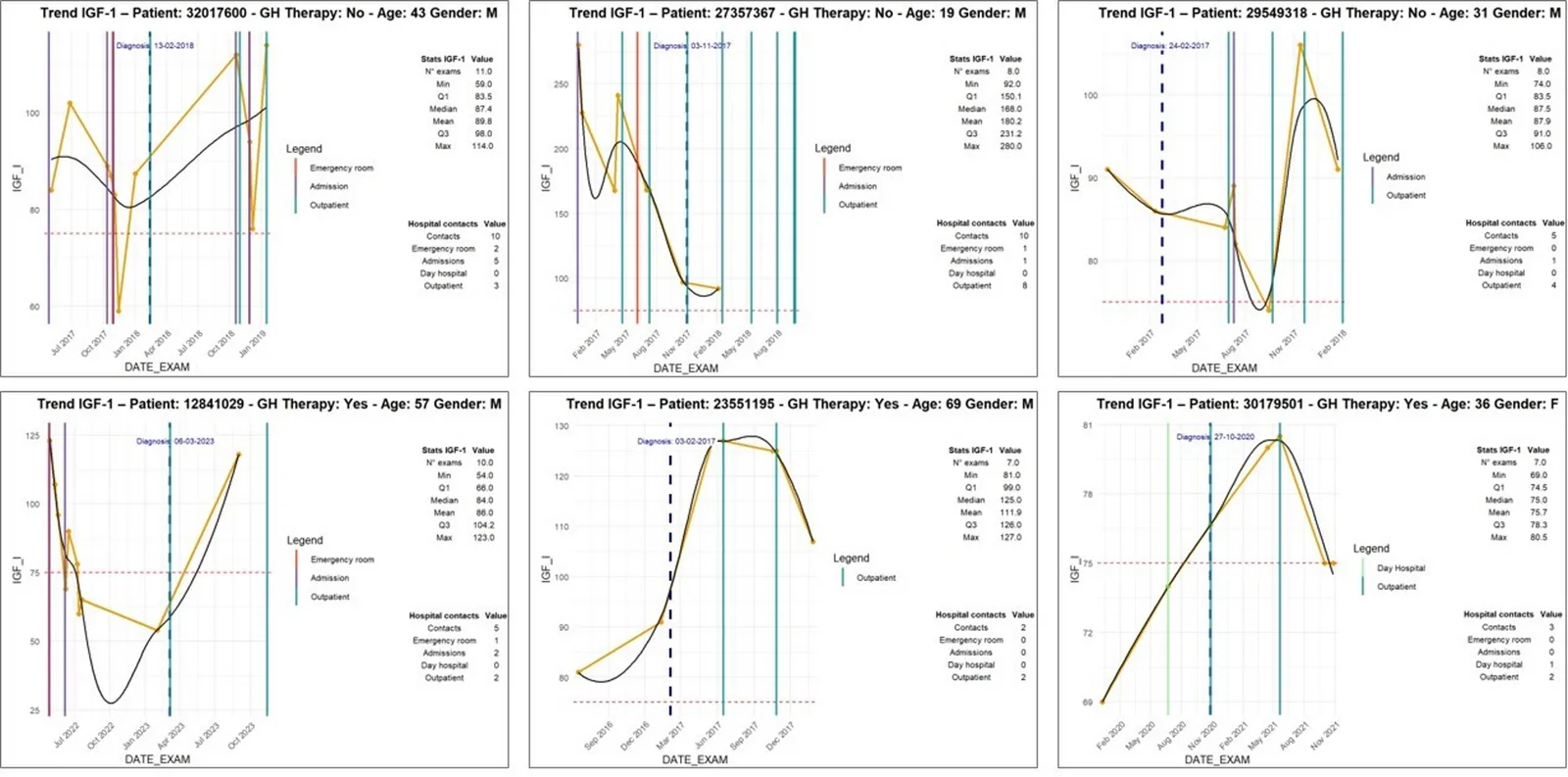
Trend evaluations

In Fig. 5, examples of trend analysis for IGF-1 levels along patient care pathways are shown for illustrative purposes, including cases in which rhGH therapy was not administered and cases in which GH replacement therapy was prescribed. These representative trajectories highlight the temporal relationship between biochemical markers, clinical events, and therapeutic decisions in patients with suspected or confirmed AGHD. The visualization integrates major clinical milestones, including the timing of diagnosis and different types of healthcare encounters (hospitalizations, emergency visits, day hospital admissions, and outpatient visits), represented as vertical reference lines combined with smoothed trend curves. This combined representation allows for a comprehensive assessment of how IGF-1 dynamics evolve in relation to both diagnostic confirmation and subsequent healthcare utilization. Overall, the trend-based evaluation provides an intuitive and clinically meaningful framework to contextualize IGF-1 measurements over time, supporting the interpretation of biochemical patterns in relation to treatment exposure and care pathways. This approach complements cross-sectional analyses by capturing longitudinal changes and heterogeneity at the individual patient level.

### Final validation

The validation process took place at multiple stages of data extraction and transformation, supported by standardized reporting procedures. As the work relied mainly upon unstructured data required, a thorough two-step validation was provided: an initial technical verification of the extracted concepts, followed by an independent clinical review. These iterative evaluations were essential for refining and stabilizing the text-mining rules used in the NLP workflows. During the final validation phase, the complete dataset underwent an additional layer of quality checks to ensure coherence across all variables and data sources [25]. Particular attention was given to variables expected to maintain logical relationships (such as BMI and obesity), which were re-examined to confirm internal consistency after consolidation. This last step allowed us to verify that the integrated dataset accurately reflected clinical reality and was suitable for subsequent analysis within the AGHD Data Mart.

## Discussion

The AGHD Data Mart represents one of the earliest practical applications of AI within the clinical context of AGHD. In the near future, a broader and more systematic use of AI in this field is expected, particularly through the exploitation of large-scale data to enable ML algorithms for disease phenotyping and therapeutic personalization [26]. However, such developments necessarily rely on the solid construction of the lower levels of the AI-driven evidence pyramid. At its foundation lie hospital information technologies infrastructures, capable of collecting reliable and secure data from multiple sources and their aggregation within data warehouses, and subsequently the definition of disease-specific Data Marts [27]. This perspective has guided the present work.

Given its primary objective of assessing the feasibility of building an AGHD Data Mart, the study should be considered methodological in its nature. Nonetheless, selected disease-related descriptive endpoints were included with the aim of demonstrating to the endocrinology community the practical value of an AI-mediated data collection system, rather than providing definitive epidemiological or clinical inferences.

For data extraction and cohort identification within the hospital data warehouse, we relied on a combination of ICD-9–based diagnostic criteria [28], currently used in Italy for reimbursement purposes, and NLP-based analysis of outpatient clinical reports. One of the first challenges encountered relates to the ICD coding system itself. Unlike ICD-11 [29], which assigns a single, unique code to adult GHD, ICD-9 includes multiple codes potentially referring to this condition. This lack of specificity made ICD-9–based extraction useful, but less efficient than text-mining approaches aimed at identifying AGHD-related terminology within clinical narratives. Of the 210 initially selected patients, 11.5% were excluded following a detailed review of individual clinical documentation by the endocrinology team. This finding highlights both the imperfection of the methodology and, at the same time, the relatively limited error rate of the overall approach [30].

Two diagnostic modalities were recognized in accordance with current guidelines: the presence of a dynamic test compatible with GHD or the coexistence of anterior panhypopituitarism with subnormal IGF-1 levels [2, 24]. When neither criterion could be clearly identified through report analysis or review of the EMR, the diagnostic modality was classified as “AI-based.” It is important to highlight that this last definition reflects a case identification strategy rather than an AI-generated diagnostic adjudication and refers to patients in whom AGHD was explicitly reported in clinical documentation despite the absence of structured baseline diagnostic data. This typically occurred in cases where objective information, such as IGF-1 values at T0 or results of dynamic testing, was unavailable.

Teaching AI systems to identify IGF-1 values below − 2 SDS would require a more advanced LLM-based approach, given the need to integrate absolute IGF-1 levels with patient age and sex (and according to the assay). To address this limitation, an absolute IGF-1 threshold of 75 ng/mL was adopted as an operational surrogate to define subnormal values, according to 5th percentile of a population with median age 51 years ​​ [31] and according to the main reference interval of several assays independently from gender ​ [32]. While this choice represents a limitation of the study, it was not intended as a diagnostic criterion but rather as a pragmatic solution for data harmonization, given the fact that data came from the real world. Given the age distribution of our cohort, this cutoff was considered a reasonable discriminator in patients with panhypopituitarism identified through text mining, therapy records, and laboratory data. Among the 188 validated patients, a good cohort compared with some European series [33], 28.2% were diagnosed by dynamic testing, 54.3% by the combination of anterior panhypopituitarism and low IGF-1, and 17.6% through the AI-assisted approach (Fig. 4). As reported previously, this last group refers to patients with clearly stated AGHD diagnosis on outpatient reports, but with no results of dynamic tests or IGF-1 plus information on anterior panhypopituitarism available in the EMR. It is presumable, thus, that the true percentage of patients who had been diagnosed through dynamic tests is higher than the one reported in the Data Mart. Importantly, the entire electronic medical records, including all patient contacts from the first access (T0), were reviewed to define the diagnostic modality.

Etiological assessment represented another key component of the study. NLP techniques were extensively applied to T0 reports using disease-specific terms defined by the endocrinology team. This strategy allowed recovery of etiological information in 87.8% of cases, with only 12.2% remaining unspecified. All AI-derived results were subsequently reviewed by clinicians. In line with major population registries, post-surgical etiology emerged as the most frequent cause of AGHD. The proportion of idiopathic cases was comparable to that reported in other registries (7% vs. 4–24%), whereas empty sella syndrome appeared more prevalent in our cohort, representing a distinctive feature compared with other series [34]. Direct comparisons with international registries should be interpreted with caution, given differences in inclusion criteria, healthcare organization, and regulatory frameworks.

Only 37.8% of patients in our AGHD population were receiving rhGH therapy, a proportion lower than that reported in several European centers [33]. This finding may be, in our opinion, explained by the Italian regulatory framework (AIFA Note 39), which links reimbursement eligibility strictly to the presence of a positive dynamic test. Although anterior panhypopituitarism with low IGF-1 is recognized as a diagnostic criterion by current guidelines, it does not grant economic coverage for rhGH therapy within the national health system. Furthermore, the recent unavailability of GHRH, commonly used in combination with arginine for dynamic testing in Italy, has further reduced the number of tests performed in recent years [35].

Interestingly, there was a trend toward statistical significance for a lower prevalence of diabetes mellitus among patients treated with rhGH. This finding may highlight a potential management pitfall, as clinicians may be more reluctant to initiate rhGH in patients with metabolic comorbidities. Since type 2 diabetes mellitus is not an absolute contraindication to rhGH (unlike active diabetic retinopathy) [36], this AI-driven observation offers an opportunity to reassess prescription practices and improve alignment with current recommendations.

With regard to biochemical data, whether retrieved as structured information from in-house laboratory testing or as unstructured data from clinical reports, a substantial degree of missingness was observed. This underscores the need for standardization of laboratory parameters both in terms of clinical practice and of results to be recorded in outpatient reports, regardless of the individual physician [37]. Notably, NLP-based approaches could support the development of an electronic case report form without requiring direct implementation at the system level.

For etiological and biochemical analyses, data were anchored to T0, the first contact at our institution, along with any additional contacts within the subsequent 365 days. Longitudinal analysis across multiple patient encounters represents a natural next step in the evolution of this project. For the present paper, we performed a preliminary longitudinal exploration of patient-specific biochemical patterns . Within the Data Mart, such analyses, potentially updated in real time, may allow continuous tracking of clinical trajectories. For instance, a patient receiving rhGH therapy but showing a progressive decline in IGF-1 levels may raise suspicion of poor adherence [6]. An alert-based system, including digital monitoring tools, could help provide targeted clinical support and adherence interventions [38]. This approach aligns with the broader objective of precision medicine and individualized therapy in AGHD, while being considered at the present time as a clinical support framework rather than a decision-making replacement.

This study has a number of limitations that should be acknowledged. As its primary aim was methodological, the development of an AI-driven AGHD Data Mart, the clinical accuracy of some results cannot be considered definitive, despite careful medical review. Relevant limitations include the operational management of IGF-1 values without full SDS normalization, the presence of missing biochemical data, incomplete etiological attribution in a subset of patients, and heterogeneity in the documented diagnostic pathways. These aspects are partly related to real world data themselves [13, 39] ​​and partly to the intrinsic characteristics of AGHD [40], a condition with subtle clinical expression, mainly managed in the outpatient setting, and lacking a single, unambiguous ICD-9 code within the Italian healthcare system. From this perspective, the complexity encountered reflects the real-world nature of the disease rather than methodological weakness of the study alone. The present approach is not intended to replace established population registries, which remain essential for standardized and curated evidence generation. Instead, it proposes a complementary, AI-supported framework for the structured retrieval of real-world clinical data. In the future, further methodological refinement may allow the integration of additional unstructured elements, such as narrative clinical information or individualized management considerations, that are currently beyond the scope of this work. Building on the standardized and scalable Data Mart infrastructure, future work will focus on expanding AI-based analyses within the AGHD domain. Planned developments include the implementation of predictive and exploratory AI models, such as NLP-based tools for information extraction and algorithms for phenotypic clustering. In parallel, an early LLM prototype trained on a limited subset of clinical records will be tested to assess feasibility, vocabulary specificity, and data structure. This pilot will explore automated validation of quantitative data, treatment pathways, and dosing patterns, while supporting more accurate interpretation of unstructured clinical information. These steps will inform the design of future, disease-specific LLMs integrated within the AGHD Data Mart.

In conclusion, this study should be regarded as a foundational step toward the cautious integration of AI into AGHD research, paving the way for the development of reliable, center-specific AI tools aimed at supporting data analysis and, ultimately, more personalized patient management.

## Appendix 1 - Study data model (Ontology)

**Figure:**